# A Retrospective Study of Central Line-Associated Bloodstream Infections in Children Treated With Hemodialysis in a Tertiary Care Center

**DOI:** 10.7759/cureus.77143

**Published:** 2025-01-08

**Authors:** Abdullah T Al Qahtani, Norah AlSumih, Soud A Al Rasheed, Abdulrahman Alamir

**Affiliations:** 1 Department of Pediatric Nephrology, King Abdullah Specialist Children's Hospital, Riyadh, SAU; 2 Department of Pediatric Nephrology, King Abdullah Specialist Children’s Hospital, Riyadh, SAU; 3 Department of Pediatric Nephrology, Ministry of National Guard Health Affairs, King Abdulaziz Medical City, Riyadh, SAU

**Keywords:** catheter-associated bacteremia, catheter-related bacteremia, central-line associated bloodstream infections, dialysis catheter, end-stage renal disease (esrd), general nephrology dialysis and transplantation, hd (hemodialysis), hemodialysis access, tunneled dialysis catheter

## Abstract

Background

Central venous catheter (CVC) poses a significant risk of infectious complications in children undergoing hemodialysis. A major concern is the occurrence of central line-associated bloodstream infections (CLABSIs), which are most critical and lead to significant morbidity.

Methods

We conducted a study in our pediatric hemodialysis center in King Abdullah Specialized Children’s Hospital, Riyadh, Saudi Arabia. We included pediatric patients (younger than 14 years old) with a positive central line blood culture who underwent hemodialysis from 2015 until 2023. We collected data pertaining to age, sex, underlying disease, clinical manifestations, and microbiological features, as well as their management strategies. We compared this group’s epidemiological and biochemical markers to a group of hemodialysis patients who did not develop CLABSI.

Results

In our pediatric hemodialysis center, we have an overall incidence of 4.2 CLABSIs per 1,000 catheter days, with mainly gram-positive organisms. We found that more than half of the patients required hospitalization, indicating a lower threshold for admission in pediatric patients than adults. We also found that patients with catheter infections had higher white blood cell counts as well as neutrophils, which can be used to raise suspicion of catheter infection. Most patients (85%) did not require catheter removal and were treated with intravenous antibiotics only.

Conclusion

CLABSIs cause high morbidity and mortality; therefore, clinicians should have a high index of suspicion, especially in patients with fever and a high white blood cell count. Such patients require intravenous antibiotics, lock therapy in some cases, and, occasionally, catheter removal/replacement as indicated.

## Introduction

An effective vascular access is essential for successful hemodialysis (HD). The central venous catheter (CVC) poses a significant risk of infectious complications in children undergoing HD [1]. One of the primary concerns is the occurrence of central line-associated bloodstream infections (CLABSIs), which are most critical and cause significant morbidity [2]. CLABSIs often arise from the colonization of the catheter's external surface, typically caused by the migration of skin organisms.

Rates of CLABSI exhibit significant variability, influenced by factors such as the size of the hospital and the specific service or unit within the hospital. In most pediatric and adult studies, the reported overall rate of CLABSI ranged from 1.2 to 1.6 episodes per 1,000 catheter days [3-6].

Over the past decade, a significant concern has been the rising prevalence of antimicrobial-resistant micro-organisms in dialysis facilities. Recent data indicate that methicillin-resistant *Staphylococcus aureus* (MRSA) was identified in 20%-40% of bacteremic episodes in HD patients [6].

As per the latest guidelines, CLABSIs are defined as positive blood culture from an intravascular catheter, along with at least one positive blood culture collected from a peripheral vein (if possible) [7-8]. Clinical symptoms of infection, such as fever, chills, and/or hypotension, should also be present, and there should be no evident source of infection other than the catheter [9-10].

The occurrence of CLABSI in children undergoing HD has been explored in only a limited number of studies. Therefore, the approach to managing infections related to CVCs in this population primarily relies on information gained from adult studies.

This study explores CLABSI in children undergoing HD in a tertiary center, including their epidemiological, microbiological, and biochemical features, as well as management and complications. We will compare data and outcomes between a group of HD patients with CLABSI against HD patients without CLABSI to uncover risk factors for developing catheter-related infections.

## Materials and methods

Specific objectives 

The main objectives of this study were to evaluate the epidemiological, microbiological, and biochemical features of CLABSI in children undergoing HD in a tertiary center, describe the different managements employed for CLABSI in children undergoing HD in a tertiary center and their recurrence and complications, and compare data and outcomes between a group of HD patients with CLABSI with HD patients without CLABSI to uncover risk factors for developing catheter-related infections.

Study area/setting 

The study was conducted in King Abdullah Specialized Children’s Hospital, Riyadh, Saudi Arabia, which is a tertiary governmental hospital with a bed capacity of 320 beds designated for general pediatrics and pediatric subspecialties. This hospital has a dedicated HD unit for pediatric patients. 

Study subjects 

After obtaining IRB approval, data were retrieved from the hospital’s digital medical records of all patients who developed CLABSI while undergoing HD from 2015 until 2023 and met the following inclusion and exclusion criteria.

Inclusion Criteria

The study included all pediatric patients (<14 years) treated with HD with positive central-line blood cultures. 

Exclusion Criteria

The study excluded peripheral line bacteremia in patients undergoing HD with negative central-line blood culture. Exit-site and tunnel infections with negative central-line blood culture were also excluded. 

Study design 

This is a retrospective cohort study.

Data collection and management plan 

Data were obtained as a chart review from the medical records of all patients undergoing HD in our center from 2015 until 2023, which is in total 39 patients. These patients were divided into two groups: CLABSI group and non-CLABSI group. The study included demographics, primary disease, type of catheter, and days on catheter. We also investigated microbiological characteristics of these CLABSIs, in addition to biochemical markers and management courses. 

Statistical analysis 

SPSS software Version 23.0 (IBM Corp., Armonk, NY, USA) was used for statistical analysis. Mean and standard deviation were used for quantitative variables. Qualitative variables were expressed as percentages and frequencies. Comparison between quantitative variables was made using the Mann-Whitney U test, and Fisher's exact test was used to assess the association between categorical variables. A p-value was considered significant if it was less than 0.05.

## Results

Characteristics of participants

Our records showed that 39 patients underwent HD in our unit from 2015 until 2023. A total of 18 pediatric patients out of those 39 patients with HD catheters were included in this study, and the rest were excluded mainly due to age being older than 14 years at the time of the study. Seven (38.9%) patients had positive central line blood cultures. We will refer to them as the CLABSI group, while the remaining 11 will be referred to as the no CLABSI group. Ten (55.6%) were females and 8 (44.4%) were males. Majority of the CLABSI group were females (85.7%), while most of the no CLABSI group were males (63.6%). The average age of the participants was 10.4 ± 3.0 years (range: 3.5 to 14 years). The mean age of the CLABSI group was 10.9 ± 2.4 years, and the average age of no CLABSI group was slightly lower (9.7 ± 3.6 years). 

Etiology of participants

Regarding the primary disease, hypoplastic kidneys and obstructive uropathy were the most reported diseases (33.3% for each), followed by bilateral Wilm’s tumor (11.1%). Hypoplastic kidney was most reported among the CLABSI group (42.9%), while obstructive uropathy was most reported among the no CLABSI group (36.4%) (Table 1).

**Table 1:** Characteristics of study participants. ARPKD, autosomal recessive polycystic kidney disease; HUS, hemolytic uremic syndrome

Variable	Categories	CLABSI group (n=7)	No CLABSI group (n=11)	Total (n=18)
Age (in years)	Mean ± SD (range)	10.9 ± 2.4 (5 - 14)	9.7 ± 3.6 (3.5 - 14)	10.4 ± 3.0 (3.5 – 14)
Gender	Male	1 (14.3)	7 (63.6)	8 (44.4)
	Female	6 (85.7)	4 (36.4)	10 (55.6)
Primary disease	Hypoplastic kidneys	3 (42.9)	3 (27.3)	6 (33.3)
	Obstructive uropathy	2 (28.6)	4 (36.4)	6 (33.3)
	Bilateral Wilms’ tumor	1 (14.3)	1 (9.1)	2 (11.1)
	Joubert syndrome	1 (14.3)	0 (0)	1 (5.6)
	ARPKD	0 (0)	1 (9.1)	1 (5.6)
	Atypical HUS	0 (0)	1 (9.1)	1 (5.6)
	Unknown	0 (0)	1 (9.1)	1 (5.6)

**Figure 1:**
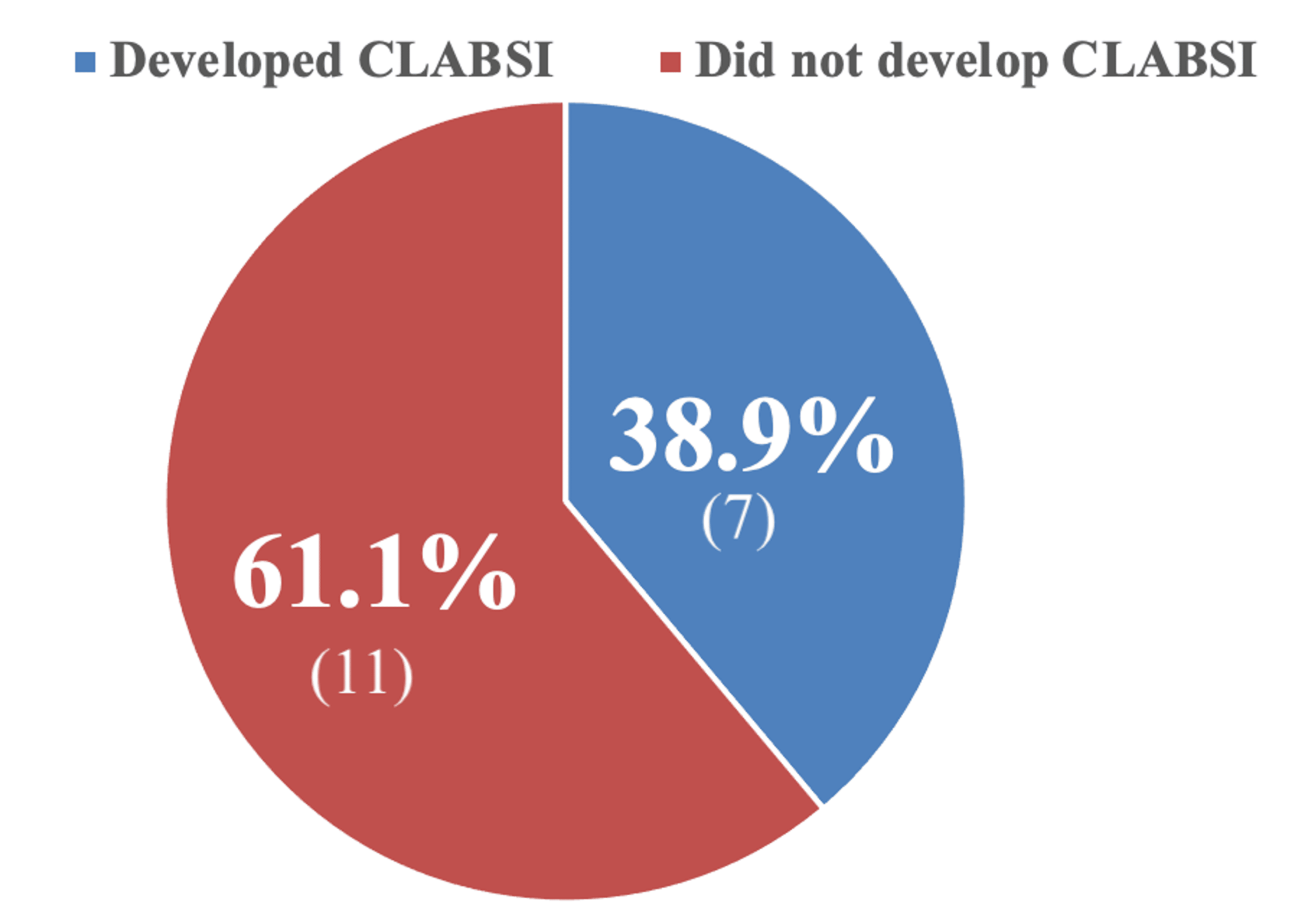
Prevalence of CLABSI in HD patients. CLABSI, central line-associated bloodstream infection; HD, hemodialysis

Prevalence of CLABSI in children treated with HD was found to be 38.9% (n=7) (Figure 1).

Clinical manifestations of CLABSI 

Regarding the CLABSI group (n = 7), most of the patients had positive central line blood culture more than once, which came to a total of 14 cases of CLABSI. Concerning the presenting symptoms or signs, fever or chills was the most reported symptom, which was experienced by all patients (100%), followed by nausea and vomiting (14.3%) and then pain at catheter site/redness/discharge, loose stool, and abdominal pain (7.1% for each). The CLABSI group had positive central blood culture, and only two (14.3%) episodes showed positive peripheral blood culture as well taken at the same time.

Microbiology of CLABSI 

Regarding organism isolated from central blood culture, *Enterobacter cloacae* (21.4%) was the most isolated bacterium, followed by *Pseudomonas aeruginosa*, *Staphylococcus aureus*, *Staphylococcus warneri*, and *Streptococcus viridans *group (14.3% for each), and there was no polymicrobial isolate. Resistance against antibiotics was reported in three (21.4%) of the cases mainly against clindamycin in two cultures, and isolated organism was Staphylococcus warneri for both. We also isolated one case of MRSA that was resistant to cefazolin, oxacillin, and gentamicin. 

Eight (57.1%) of the cases were hospitalized and none needed intensive care. Intravenous (IV) antibiotics were used for all patients as management for CLABSI (100%), followed by lock therapy (21.4%) and catheter exchange (14.3%). CLABSI reoccurred within six weeks in two (14.3%) of the cases, and there were no mortality cases (Table 2).

**Table 2:** Clinical manifestations and microbiology of CLABSI. CLABSI, central line-associated bloodstream infection

Variable	Categories	N (%)
Presenting symptoms/signs	Fever/chills	14 (100)
	Nausea and vomiting	2 (14.3)
	Pain at catheter site/redness/discharge	1 (7.1)
	Loose stool	1 (7.1)
	Abdominal pain	1 (7.1)
Positive central blood culture	Yes	14 (100)
	No	0 (0)
Positive peripheral blood cultures (at the same time)	Yes	2 (14.3)
	No	12 (85.7)
Organism isolated from central cultures	Enterobacter cloacae	3 (21.4)
	Pseudomonas aeruginosa	2 (14.3)
	Staphylococcus aureus	2 (14.3)
	Staphylococcus warneri	2 (14.3)
	*Streptococcus viridans *group	2 (14.3)
	Methicillin-resistant *Staphylococcus aureus*	1 (7.1)
	Acinetobacter baumanni	1 (7.1)
	*Streptococcus pyogenes *(group A)	1 (7.1)
Antimicrobial resistance	None	11 (78.6)
	Clindamycin	2 (14.3)
	Cefazolin	1 (7.1)
	Oxacillin	1 (7.1)
	Gentamicin	1 (7.1)
Polymicrobial cultures	No	14 (100)
Hospitalization	Yes	8 (57.1)
	No	6 (42.9)
Intensive care	Yes	0 (0)
	No	14 (100)
Management plan	Intravenous antibiotics	14 (100)
	Lock therapy	3 (21.4)
	Catheter exchange	2 (14.3)
CLABSI reoccurrence within six weeks	Recurred	2 (14.3)
	Did not recur	12 (85.7)
Mortality	Yes	0 (0)
	No	14 (100)

Comparison between the two groups

**Figure 2:**
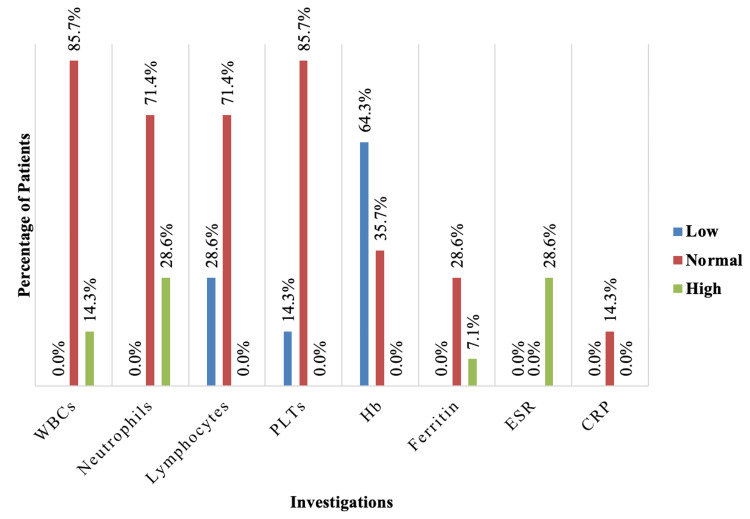
Investigations for the CLABSI group. CLABSI, central line-associated bloodstream infection; WBC, white blood cells; PLT, platelets; Hb, hemoglobin; ESR, erythrocyte sedimentation rate; CRP, C-reactive protein

When we compared the CLABSI group and the no CLABSI group, the hematological tests of CLABSI group revealed that 14.3% had leukocytosis, 28.6% had neutrophilia, 28.6% had lymphocytopenia, and 14.3% had thrombocytopenia. Anemia was reported in 64.3% of them, 7.1% had high ferritin level, and 28.6% had high erythrocyte sedimentation rate (ESR) (Figure 2).

**Figure 3:**
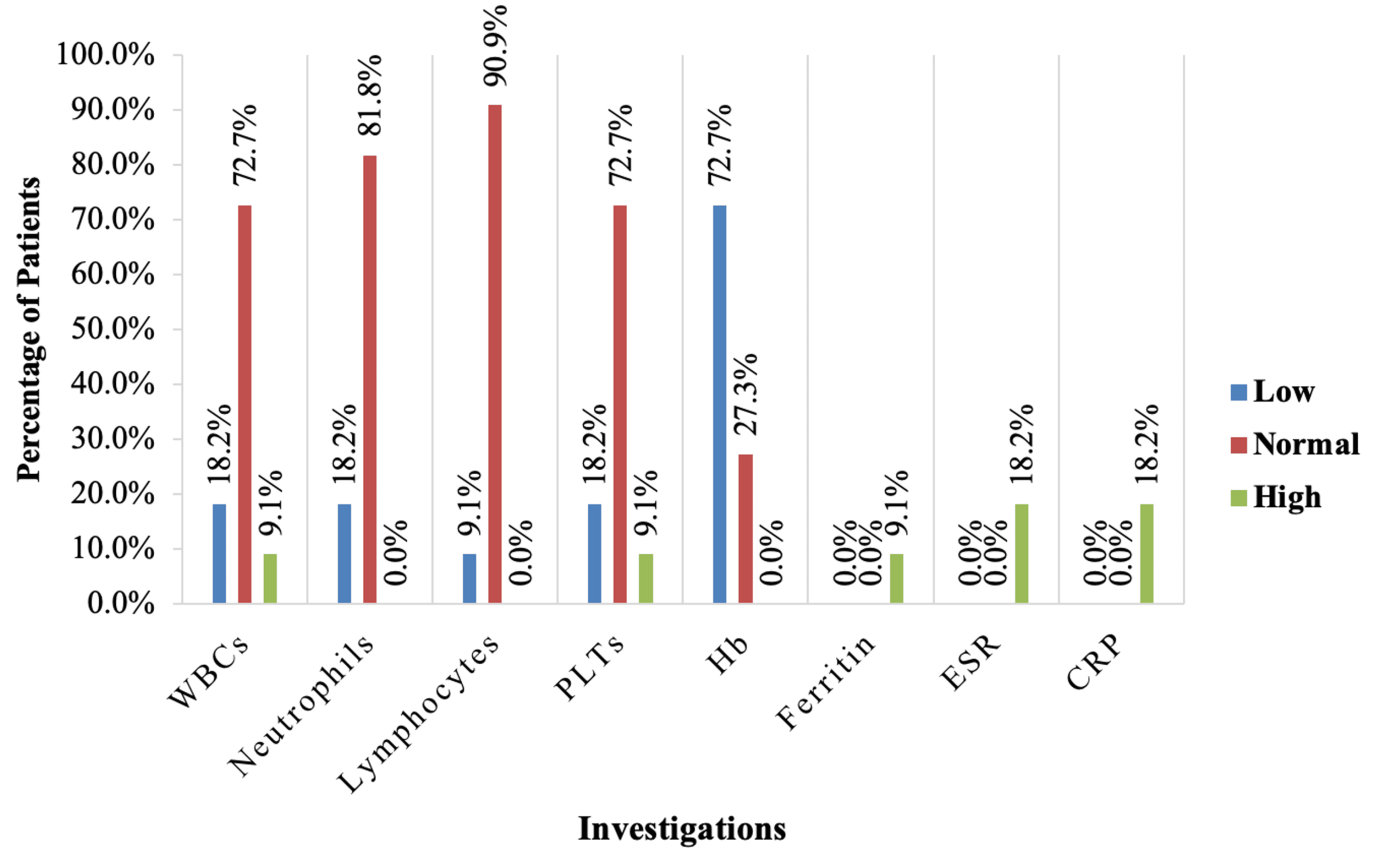
Investigations for the no CLABSI group. CLABSI, central line-associated bloodstream infection; WBC, white blood cells; PLT, platelets; Hb, hemoglobin; ESR, erythrocyte sedimentation rate; CRP, C-reactive protein

While the hematological tests of the no CLABSI group showed that 18.2% of them had leukocytopenia, 9.1% had leukocytosis, 9.1% had lymphocytopenia, 18.2% had thrombocytopenia, and 9.1% had thrombocytosis. Anemia was reported in 72.7% of them, 9.1% had high levels of ferritin, and 18.2% had high ESR and C-reactive protein (CRP) (Figure 3).

Regarding other factors and tests related to HD, the most used catheter was the tunneled catheter (96%), and the average number of days on catheter was 317 ± 348 days. All the participants had more than three HD sessions per week (100%). There was no association between type of catheter, days on catheter, and number of HD sessions per week in relation to group (p > 0.05). 

Regarding the hematological tests, the mean of white blood cells (WBCs) was 7.41 ± 3.00 x 10³/µL and the mean neutrophils was 3.82 ± 2.32 x 10³/µL, and both were significantly different between the two groups; it was higher in the CLABSI group compared to the no CLABSI group (P = 0.007 and 0.005 respectively). 

The average number of lymphocytes was 2.06 ± 0.93 x 10³/µL and for platelets was 234 ± 91 x 10³/µL. The average hemoglobin level was 108 ± 16 g/L, and the average ferritin level was 121.3 ± 100.7 ng/mL. The mean ESR and CRP were found to be 49.7 ± 25.1 mm/hr and 42 ± 73 mg/L, respectively. There was no difference between the CLABSI group and the no CLABSI group in terms of other factors (p > 0.05) (Table 3).

**Table 3:** Hemodialysis factors its association with the CLABSI group and the no CLABSI group. ^F^The p-value was calculated using Fisher's exact test, and other p-values were calculated using the Mann-Whitney U test. *Significant p-value < 0.05. CLABSI, central line-associated bloodstream infection; WBC, white blood cells; PLT, platelet; ESR, erythrocyte sedimentation rate; CRP, C-reactive protein

Variable	Categories	CLABSI group	No CLABSI group	Total	p-Value
Type of catheter	Tunneled	14 (100)	10 (90.9)	24 (96)	0.440^F^
	Untunneled	0 (0)	1 (9.1)	1 (4)	
Days on catheter	Mean ± SD	256 ± 251	396 ± 444	317 ± 348	0.443
Number of hemodialysis sessions per week	>3	14 (100)	11 (100)	25 (100)	-
WBCs (x10³/µL)	Mean ± SD	8.74 ± 2.56	5.72 ± 2.72	7.41 ± 3.00	0.007*
Neutrophils (x10³/µL)	Mean ± SD	4.93 ± 2.40	2.41 ± 1.26	3.82 ± 2.32	0.005*
Lymphocytes (x10³/µL)	Mean ± SD	1.91 ± 1.01	2.24 ± 0.81	2.06 ± 0.93	0.352
PLTs (x10³/µL)	Mean ± SD	238 ± 77	229 ± 111	234 ± 91	0.661
Hemoglobin (g/L)	Mean ± SD	111 ± 10.0	103 ± 20.6	108 ± 16	0.528
Ferritin	Mean ± SD	92.2 ± 79.4	267 ± 0	121.3 ± 100.7	0.143
ESR	Mean ± SD	39.5 ± 24.9	70.0 ± 7.1	49.7 ± 25.1	0.159
CRP	Mean ± SD	2 ± 0	82 ± 98	42 ± 73	0.102

## Discussion

CLABSI causes difficulty in all HD centers, and they are a major source of morbidity among children treated with HD. 

During a 10-year-experience study published in 2000, there were 18 episodes of CLABSI in patients undergoing HD. The average survival length of catheters was 244 days (or approximately 8 months) [11]. Previous studies have reported the rate of CLABSI to be approximately 1.2-4 CLABSI/1,000 catheter days [2,12,13]. In our pediatric HD center, the prevalence of CLABSI was found to be 38.9%, with an overall incidence of 4.2 CLABSI per 1,000 catheter days. Moreover, we had an average survival length of catheters around 317 days (or roughly 10 months). 

The presence of fever and chills in a patient with a HD catheter and no local symptoms should raise high suspicion and generally considered to be a CLABSI until proven otherwise. The clinical presentations of CLABSI can vary. However, 100% of our patients presented with fever/chills. Other manifestations included gastrointestinal upset and pain/redness at the catheter site. 

Majority of isolated bacteria were gram-positive organisms (71.4%), which is similar to the reported literature. Most patients (78.6%) did not have resistance, and we isolated one case of MRSA. We did see some resistance against clindamycin, cefazolin, oxacillin, and gentamicin, similar to the reported literature. A study conducted on a total of 68 patients with HD-related CLABSI evaluated emerging antibiotic resistance. They found that using antibiotics such as cefazolin or oxacillin as part of the initial treatment for these patients is unadvisable and recommended administering a broader spectrum antibiotic [14,15].

Peripheral blood cultures are often difficult in HD patients due to poor access; therefore, they were not sent for all patients suspected of having CLABSI. Interestingly, we were able to isolate an organism from a peripheral culture in only two of the CLABSI. However, most of existing literature does not rely on it, as it is not obtained frequently. 

Hospitalization is not required for all patients, and outpatient management is sufficient in some, as is evident in previous studies, with 37% to 47% requiring hospitalization [16,17]. Unfortunately, it was not studied in the pediatric population. For our patients, rate of hospitalization was found to be 57.1%, indicating perhaps a lower threshold for admission for the pediatric population. 

The management of CLABSI is still considered a challenge, mainly because there are issues still under debate. Several studies on children have indicated that antimicrobial therapy alone may resolve some CLABSI, potentially eliminating the need for removal of the catheter in up to 75% of cases [10]. For our patients, catheter exchange was necessary for two cases of CLABSI, both being gram-negative bacteria, whereas the rest (85.7%) were salvageable with IV antibiotics, and in three cases, lock antibiotics was used as well. 

Recurrence of CLABSI was observed in only two cases, which is sometimes attributed to bacterial adhesion to the catheter surface, creating a biofilm that causes shedding into the blood. A pediatric study found the rate of recurrence of CLABSI to be 29%. This study also found that recurrence is less likely with gram-positive organisms and after catheter removal/replacement [18]. In a cohort study including 329 adult patients, the rate of recurrence was 16.8% and was found more with gram-positive bacteria [19].

A recent study in a tertiary care hospital on adult patients with a central catheter for HD found that previous infection, high WBC count, and low hemoglobin level were independently associated with catheter-related infections [15]. Another study including adults undergoing HD found that patients with uncomplicated bacteremia had higher WBC counts and increased levels of granulocytes compared to those without bacteremia [20]. In our study, we investigated possible risk factors, especially biochemical qualities. We found that patients with catheter infections had higher WBC counts, especially neutrophils. Other parameters were also studied, including hemoglobin, platelets, ferritin, ESR, and CRP, which showed no significant difference between the two groups.

Limitations

Due to the focus on pediatric patients, the study involved a limited number of patients. In addition, some biochemical markers were not performed in some patients, which may have affected their significance as risk factors for infection. We struggled with existing literature due to a scarcity of pediatric studies on a small population such as pediatric HD patients. Therefore, further research needs to be conducted on the pediatric population.

## Conclusions

Our pediatric hemodialysis center detected 4.2 CLABSI cases per 1,000 catheter days, with predominantly gram-positive organisms (57.1%) and 21.4% antimicrobial resistance. More than half of patients were hospitalized, indicating a lower admission threshold for the pediatric population. Increased WBC count and neutrophils raise suspicion of catheter infection. Clinicians should have a higher index of suspicion of CLABSI in HD patients and ensure that gram-positive organisms are covered with appropriate antibiotics (vancomycin, piperacillin/tazobactam).
